# The Expression Profiling of the Lipoxygenase (LOX) Family Genes During Fruit Development, Abiotic Stress and Hormonal Treatments in Cucumber (*Cucumis sativus* L.)

**DOI:** 10.3390/ijms13022481

**Published:** 2012-02-22

**Authors:** Xue-Yong Yang, Wei-Jie Jiang, Hong-Jun Yu

**Affiliations:** Institute of Vegetables and Flowers (IVF), Chinese Academy of Agricultural Sciences (CAAS), Beijing, 100081, China; E-Mail: yuhj@mail.caas.net.cn

**Keywords:** lipoxygenase, cucumber, fruit development, abiotic stress, plant growth regulator

## Abstract

Lipoxygenases (LOXs) are non-haem iron-containing dioxygenases that catalyse oxygenation of polyunsaturated fatty acids and lipids to initiate the formation of a group of biologically active compounds called oxylipins. Plant oxylipins play important and diverse functions in the cells. In the current study, expression analysis during cucumber development using semi-quantitative RT-PCR revealed that 13 of 23 *CsLOX* genes were detectable, and were tissue specific or preferential accumulation. In total, 12 genes were found to be differentially expressed during fruit development and have different patterns of expression in exocarp, endocarp and pulp at day 5 after anthesis. The expression analysis of these 12 cucumber *LOX* genes in response to abiotic stresses and plant growth regulator treatments revealed their differential transcript in response to more than one treatment, indicating their diverse functions in abiotic stress and hormone responses. Results suggest that in cucumber the expanded *LOX* genes may play more diverse roles in life cycle and comprehensive data generated will be helpful in conducting functional genomic studies to understand their precise roles in cucumber fruit development and stress responses.

## 1. Introduction

Lipoxygenases (LOXs; EC 1.13.11.12) are a group of non-heme iron-containing dioxygenases that initiate the degradation of free fatty acids and esterified lipids via various branches of the LOX pathway [1]. In plants, the LOX proteins play important roles in lipid peroxidation under biotic and abiotic stress, and are involved in a number of developmental stages [2,3]. The LOX proteins catalyze the addition of oxygen to either end of a (Z, Z)-1,4-pentadiene system of polyunsaturated fatty acids to produce an unsaturated fatty acid hydroperoxide. In the case of linoleic or linolenic acids, this leads to two possible products, the 9- and 13-hydroperoxy fatty acids, which are rapidly converted either chemically or enzymatically into a group of products collectively called oxylipins, such as traumatin, jasmonic acid (JA), and methyl jasmonate (MeJA) [4]. Oxylipins have been implicated in a wide range of important physiological functions, such as signal transduction, biotic or abiotic stress response, development and senescence [5–7]. Some oxylipins are also commercially important compounds, as they can be utilized as components for fragrances [8–10].

It has been shown that, in avocados, pears and tomatoes the LOX proteins are involved in fruit development and ripening through membrane deterioration and the peroxidation of polyunsaturated fatty acids, resulting in the loss of compartmentation and cell breakdown [11–14]. In kiwifruit and peaches, LOX activity increases in conjunction with the ripening processes such as the loss of fruit firmness [15–17]. Meanwhile, some oxylipins, which are the most important compounds for fruit quality, are produced by the activity of the LOX enzymes. For instance, in the tomato, C6 alcohols and aldehydes, which are metabolized from 13-HPOs (fatty acid hydroperoxides) by the 13-LOX enzyme [18,19], constitute the major flavor volatile in the tomato; in the cucumber, the main specific aroma, (E,Z)-2,6-nonadienal (NDE), is produced by 9-LOX activity [10]. In apple, strawberry and pear, LOX activity is also reported to be associated with the production of their volatile flavors [17,20,21].

Studies on the *LOX* genes have been reported in many plant species. In *Arabidopsis thaliana*, six *LOX* genes have been already characterized enzymatically [22]. The expression of *AtLOX1* is stimulated by pathogens, abscisic acid, and methyl jasmonate [23]; *AtLOX2* is specifically involved in the biosynthesis of a jasmonate precursor [24]; *AtLOX3* and *AtLOX4* are necessary for *Arabidopsis* male fertility and flower development [25]. In the tomato, *TomloxA* and *TomloxB* are expressed in ripening tomato fruit [26,27]; *TomloxC* is not detectable until the onset of ripening and has been identified as a specific isoform involved in the generation of fatty-acid-derived flavor compounds [19,28]; *TomloxD* shows very low expression level in fruit but is rapidly induced by wounding [28]; *TomloxE* transcripts were reported to be present in breaker fruit [19]; *TomloxF* encodes a 13-LOX protein and is stimulated by *Pseudomonas putida* BTP1 [29]. Antisense techniques in the potato have shown that *PotLOX1* plays an important role in tuber development [2]; *LoxH1* is involved in the generation of volatile compounds for defense and signaling [30]; *LoxH3* is induced by wounding [31]. In *Vitis vinifera*, *VvLOXA*, *VvLOXC*, *VvLOXD* and *VvLOXO* are expressed in the grape berry and in response to pathogen infection [32]. Six *LOX* genes—*AdLox1*, *AdLox2 AdLox3*, *AdLox4*, *AdLox5* and *AdLox6*—in the kiwifruit are differentially regulated during fruit ripening and senescence [33].

In cucumbers, it has been reported that a lipid body localized LOX protein shows very early expression during cucumber seed germination [34]; a cucumber root lipoxygenase, CsLOX1 is able to act on acyl groups in phosphatidylcholine [35]; a cucumber hypocotyl-enriched *LOX* gene, *CsLOX9* was expressed *in vitro* and characterized biochemically as a 9-LOX [36]. Recently, data from cucumber genome sequencing indicate that there are 23 predicted *LOX* genes in its genome [37], and comprehensive information about gene structure and phylogeny for the entire *CsLOX* gene family are presented [38]. However, there has been no extensive molecular and expression characterization of the cucumber *LOX* gene family reported to date.

In this study, expression analyses revealed the tissue-specific or preferential accumulation of cucumber *LOX* genes, of which 12 genes were expressed differentially during fruit development and have different patterns of expression in the exocarp, the endocarp and pulp at day 5 after anthesis. The expression analyses of these 12 fruit *LOX* genes in response to abiotic stresses and plant-growth regulator treatments indicated that these genes might have differential functions in stress and hormone response, suggesting a potentially effective way to regulate LOX activity for improving fruit quality. Finally, *CsLOX2*, a type-1 LOX predicted to be 9-LOX, was suggested to participate in synthesis of cucumber flavor volatile NDE.

## 2. Results

### 2.1. The Expression Profiling of the Cucumber *LOX* Genes During Vegetative and Reproductive Development

According to the previous genome sequencing and bioinformatics analysis, the *LOX* gene family in cucumber consists of 23 *LOX* genes [37,38], of which there are nine type-1 *LOX*s predicted to be 9-LOX, which is a notable expansion in the cucumber genome (supplemental Table 1). Huang *et al*. suggested that this might be related to the flavor volatile NDE synthesis in the cucumber [37].

**Figure 1:**
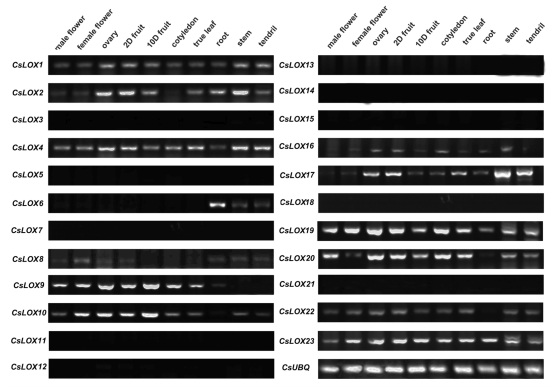
Semi-quantitative RT-PCR analysis of *LOX* genes in different tissues of cucumber (*Cucumis sativus*) cv Line 9930 (a cultivar of northern China) during development. Expressions of 23 cucumber *LOX* genes in male flower, female flower, ovary, 2 days old fruit, 10 days old fruit, cotyledon, true leaf, root, stem and tendril were analyzed. An amplified cucumber ubiquitin gene *CsUBQ* (Csa000874) was used as an internal control. The number of cycles of PCR for *LOX* genes was 30, while that for *CsUBQ* was 25.

To further study the function of *CsLOX* genes, firstly, the varied expression profiles of the 23 cucumber *LOX* family genes were analyzed during vegetative (cotyledon, true leaf, root, stem and tendril) and reproductive (male flower, female flower, ovary, 2 days old fruit (2DF) and 10 days old fruit (10DF)) development using RT-PCR (Figure 1). Primers were designed according to the sequences in the cucumber gene database. Interestingly, the results showed that 10 out of the 23 genes in the family (*CsLOX3*, *5*, *7*, *11~15, 18 and 21*) could not be detected expression in any of the ten selected tissues. Among the nine predicted type-1 LOX genes (*CsLOX1*~*6* and *8*~*10*) [38], the expression of *CsLOX1, CsLOX2*, *CsLOX4*, *CsLOX9* and *CsLOX10* could be detected in almost all ten tissues, of which *CsLOX1*, *CsLOX2* and *CsLOX4* accumulated preferentially in the developing fruit (ovary, 2DF and 10DF) and stems, while *CsLOX9* and *CsLOX10* were enriched in the developing fruit (ovary, 2DF and 10DF) and flowers (Figure 1). Interestingly, *CsLOX6* showed expression in roots, stems and tendrils, which might suggest a functional relationship within the vascular bundle. The expression of *CsLOX8* was very low, but ubiquitously expressed (Figure 1). Among the 13 predicted type-2 LOX (*CsLOX7* and *CsLOX12*~*23*) [38], *CsLOX16*, *CsLOX17*, CsLOX*19* and *CsLOX23* were all ubiquitously expressed, whereas the mRNA accumulation of *CsLOX20* and *CsLOX22* were expressed in all tissues except the roots. The expression of *CsLOX11*, which cannot be classfied in phylogenetic tree [38], coud not be detected (Figure 1).

### 2.2. The Expression Patterns of Cucumber *LOX* During Fruit Development

**Figure 2:**
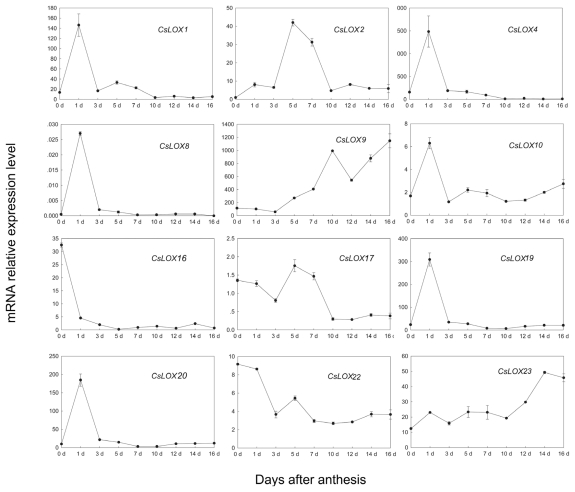
Expression profiles of *LOX* genes in cucumber (*Cucumis sativus*) cv Line 9930 (a cultivar of northern China) fruits analyzed using Quantitative Real-time PCR. The expression profiles of 12 fruit *CsLOX* genes were analyzed by using Quantitative Real-time PCR during cucumber fruit development after anthesis. *Y*-axis represents relative expression values, and the expression level of *CsLOX2* in the 0 day after anthesis was considered as 1-fold. Data represent the means ± SD of three biological replicates. The expression of all the genes was normalized with reference to the expression of *CsUBQ* gene.

Studies have shown that LOX proteins play important roles in fruit development, such as fruit ripening, senescence, softening and volatile compound formation. Our results from RT-PCR have demonstrated that the transcripts of 12 out of the 13 expressed cucumber *LOX* genes accumulated in fruit (except *CsLOX6*) (Figure 1). To study the function of *LOX* genes in cucumber fruit, we systematically analyzed their expression patterns during fruit development (before and after anthesis) by real-time RT-PCR. The expression level of *CsLOX2* on day 0 after anthesis was considered as 1-fold. As shown in Figure 2, the predicted type-1 LOX genes—*CsLOX1*, *CsLOX4* and *CsLOX9*—as well as the predicted type-2 genes—*CsLOX19* and *CsLOX20*—showed the highest expression levels during the fruit development (more than 100-fold). Among all of the 12 LOX genes, there were four expression patterns found during fruit development: I “low-high-low” (including *CsLOX1*, *CsLOX2*, *CsLOX4*, *CsLOX8*, *CsLOX10*, *CsLOX19* and *CsLOX20*), II “low-high” (including *CsLOX9* and *CsLOX23*), III “high-low” (including *CsLOX16* and *CsLOX22*) and IV “high-low-high-low” (including *CsLOX17*). Interestingly, the expression of *CsLOX* genes within pattern I also exhibited a significant difference, that was, the expression of *CsLOX1*, *CsLOX4*, *CsLOX8*, *CsLOX10*, *CsLOX19* and *CsLOX20* showed a sudden increase on 1 DAA (day after anthesis), whereas that of *CsLOX2* showed peak values from 5 to 7 DAA. This difference implied the different functions of these cucumber LOX during fruit development after anthesis. *CsLOX1*, *CsLOX4*, *CsLOX8*, *CsLOX10*, *CsLOX19* and *CsLOX20* may be involved in fruit development immediately after anthesis, such as membrane deterioration and fruit cell enlargement, while *CsLOX2* is suggested to play a role in fruit quality formation.

**Figure 3:**
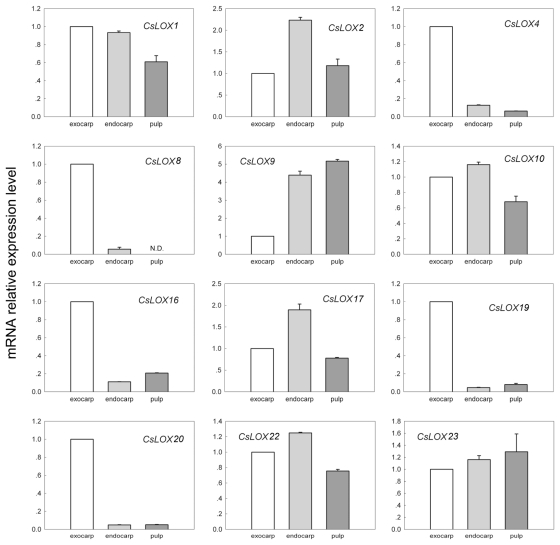
The distribution of identified 12 fruit *LOX* gene transcripts in three fruit fractions: exocarp, endocarp and pulp. The expression profiles of 12 *CsLOX* genes were analyzed by using Quantitative Real-time PCR in exocarp, endocarp and pulp of cucumber (*Cucumis sativus*) cv Line 9930 (a cultivar of northern China) fruits on 5 DAA. Data represent the means ± SD of three biological replicates. The expression of all the genes was normalized with reference to the expression of *CsUBQ* gene. N.D. represents no data.

To further understand the role of *LOX* genes in cucumber fruits, we determined the gross patterns of tissue-specific expression for each of the 12 *LOX* genes in three parts of the fruit—the exocarp, the endocarp and the pulp—on 5 DAA. The majority of *CsLOX4*, *CsLOX8, CsLOX16, CsLOX19* and *CsLOX20* transcripts were located in the exocarp, with the low amount of remaining expression distributed between the endocarp and the pulp (Figure 3), which suggested they might function in exocarp development and compounds synthesis for resisting fungi and pest. The opposite was observed for two type-1 LOX, *CsLOX2* and *CsLOX9*, the transcripts of which were found to mainly localize in the endocarp and the pulp (Figure 3). Transcripts of *CsLOX17* were found predominantly in the endocarp, with the remaining expression split equally between the exocarp and the pulp (Figure 3). The transcripts of *CsLOX1*, *CsLOX10*, *CsLOX22* and *CsLOX23* were found to be split relatively evenly among all compartments (Figure 3).

### 2.3. The Expression of Fruit *CsLOX* Genes in Response to Plant Hormones and Abiotic Stress Treatments

**Figure 4:**
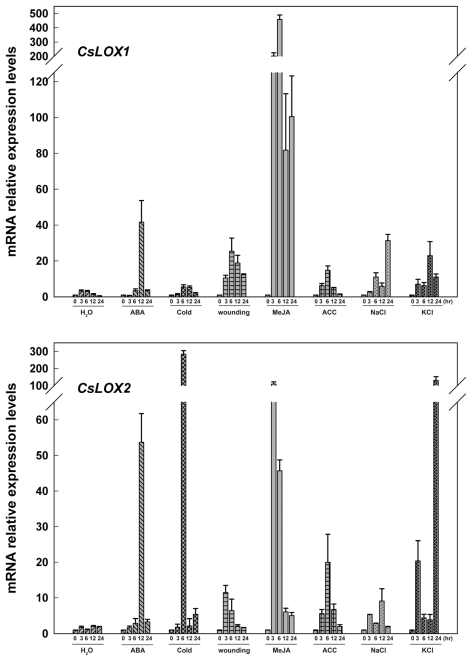
Expressions of *CsLOX1* and *CsLOX2* in cucumber leaves at various time points under treatments with H_2_O, ABA (100 μM), cold (4 °C), wounding, MeJA (100 μM), ACC (100 μM) NaCl (200 mM) and KCl (200 mM) were analyzed by Real-time PCR. A cucumber actin gene *CsACT* (Csa017310) was detected as internal reference. Data represent the mean ± SD of three independent biological determinations.

**Figure 5:**
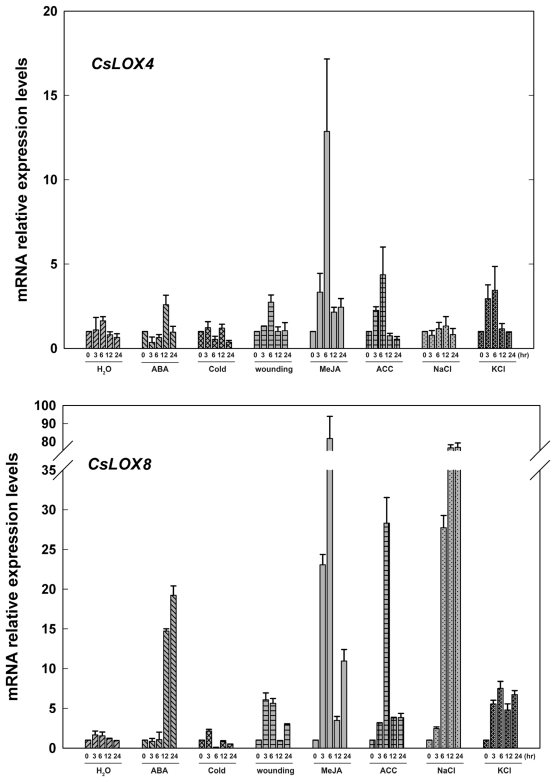
Expressions of *CsLOX4* and *CsLOX8* in cucumber leaves at various time points under treatments with H_2_O, ABA (100 μM), cold (4 °C), wounding, MeJA (100 μM), ACC (100 μM) NaCl (200 mM) and KCl (200 mM) were analyzed by Real-time PCR. A cucumber actin gene *CsACT* (Csa017310) was detected as internal reference. Data represent the mean ± SD of three independent biological determinations.

**Figure 6:**
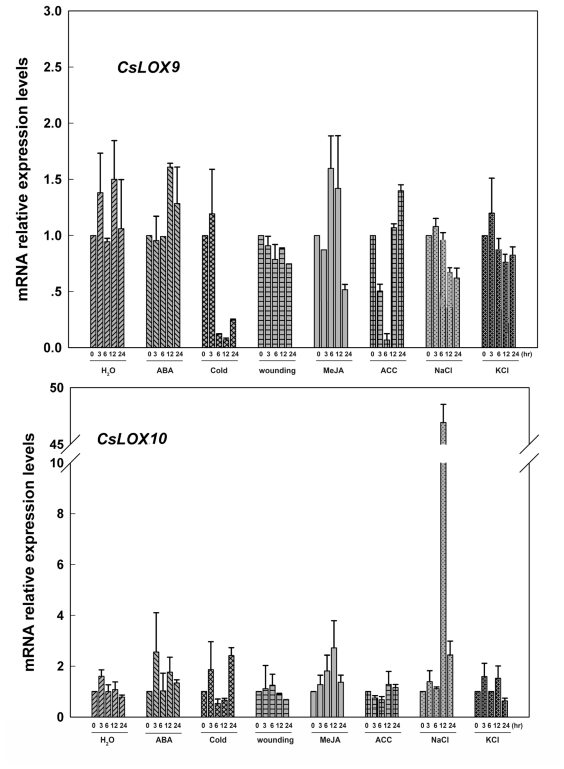
Expressions of *CsLOX9* and *CsLOX10* in cucumber leaves at various time points under treatments with H_2_O, ABA (100 μM), cold (4 °C), wounding, MeJA (100 μM), ACC (100 μM) NaCl (200 mM) and KCl (200 mM) were analyzed by Real-time PCR. A cucumber actin gene *CsACT* (Csa017310) was detected as internal reference. Data represent the mean ± SD of three independent biological determinations.

**Figure 7:**
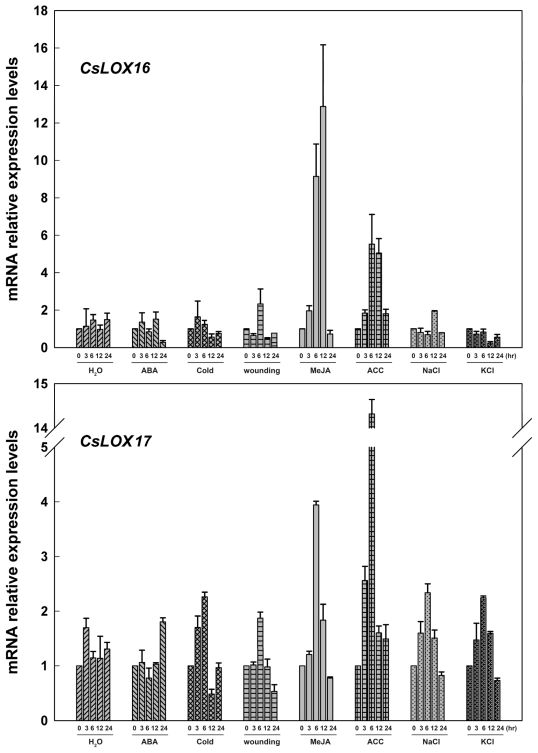
Expressions of *CsLOX16* and *CsLOX17* in cucumber leaves at various time points under treatments with H_2_O, ABA (100 μM), cold (4 °C), wounding, MeJA (100 μM), ACC (100 μM) NaCl (200 mM) and KCl (200 mM) were analyzed by Real-time PCR. A cucumber actin gene *CsACT* (Csa017310) was detected as internal reference. Data represent the mean ± SD of three independent biological determinations.

**Figure 8:**
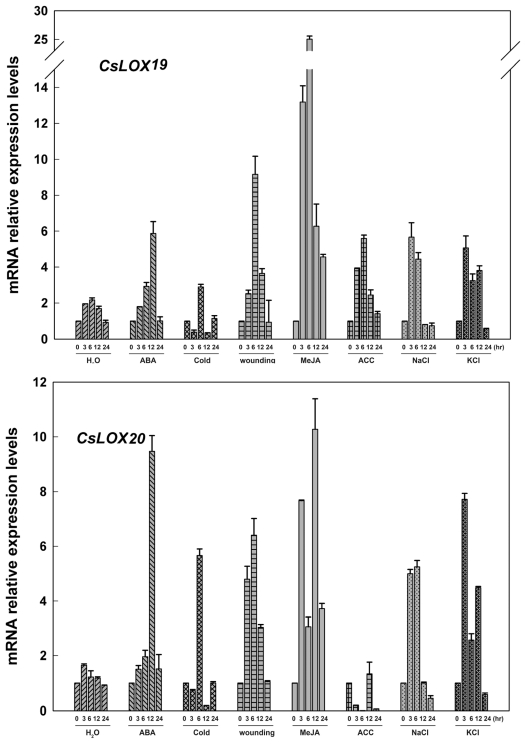
Expressions of *CsLOX19* and *CsLOX20* in cucumber leaves at various time points under treatments with H_2_O, ABA (100 μM), cold (4 °C), wounding, MeJA (100 μM), ACC (100 μM) NaCl (200 mM) and KCl (200 mM) were analyzed by Real-time PCR. A cucumber actin gene *CsACT* (Csa017310) was detected as internal reference. Data represent the mean ± SD of three independent biological determinations.

**Figure 9:**
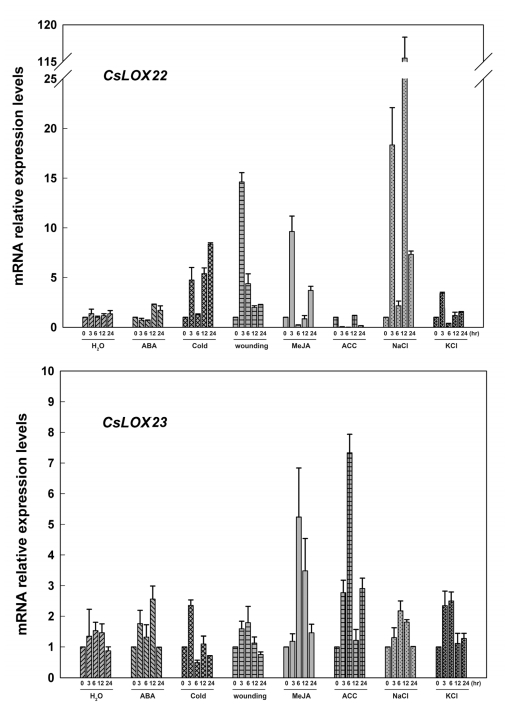
Expressions of *CsLOX22* and *CsLOX23* in cucumber leaves at various time points under treatments with H_2_O, ABA (100 μM), cold (4 °C), wounding, MeJA (100 μM), ACC (100 μM) NaCl (200 mM) and KCl (200 mM) were analyzed by Real-time PCR. A cucumber actin gene *CsACT* (Csa017310) was detected as internal reference. Data represent the mean ± SD of three independent biological determinations.

LOX proteins and their metabolites have been demonstrated to be involved in plant defense responses during various stresses [4,6,7]. In addition, abscisic acid (ABA), methyl jasmonate (MeJA) and ethylene are well-known modulators of defense responses in plants. To study cucumber *LOX* gene expressions in response to plant hormones and abiotic stress, and to explore potential ways to alter transcription for improving fruit quality, the expressions of the 12 fruit *LOX* genes in cucumber leaves at the two-leaf stage were analyzed in response to ABA (100 μM), cold (4 °C), wounding, MeJA (100 μM), 1-aminocyclopropane-l-carboxylic acid (ACC; precursor of ethylene) (100 μM), NaCl (200 mM) and KCl (K^+^ is also a nutrient element; 200 mM) by Real-time PCR with H_2_O treatment as a control. As shown in Figures 4–9, all of the analyzed genes exhibited differential accumulation or downregulation in response to at least one treatment.

Among the 12 fruit *LOX* genes, six (*CsLOX1*, *2*, *4*, *8*, *9* and *10*) are predicted to be type-1 LOXs, of which *CsLOX2* transcripts were transiently induced by exogenous MeJA and reached a maximum of up to a 100-fold at 3 hr after treatment (Figure 4). Similarly, wounding, which is known to induce the endogenous accumulation of JA [39], had the same effect on *CsLOX2* mRNA, with a maximum accumulation at 3 hr, but it was reduced effect compared to that induced by MeJA (Figure 4). Available evidence suggests that MeJA and ethylene usually act synergistically in defense pathways, likely via the involvement of the ERF1 transcription factor [40–42]. In our study, mRNA levels of *CsLOX2* reached a maximum of up to 20-fold at 6 hr after treatment with ACC (Figure 4). *CsLOX2* was also induced by ABA, with mRNA accumulation reaching the maximal levels up to 50-fold at 6 hr after treatment. ABA was shown to be involved in cold and salt stress responses, the study on the accumulation of *CsLOX2* mRNA in response to low temperature, NaCl and KCl showed that the transcripts were transiently induced by cold and reached a maximum at 12 h (almost 300-fold), and were largely induced and reached two peak values at 3 h (20-fold) and 24 h (100-fold) by KCl. *CsLOX2* transcripts were also inducible by NaCl, but the effect was not comparable to that of cold and KCl (Figure 4). It is worthy of note that K^+^ treatment not only affects osmotic stress, but K^+^ is also a necessary nutrient for improving fruit quality, such as flavor, in many plant species [43,44]. Considering the expression pattern of *CsLOX2* during fruit development, the results suggested that *CsLOX2* might be involved in flavor synthesis in cucumber.

For *CsLOX1*, exogenous JA and wounding both induced steady-levels of its mRNA accumulation starting from 3 h to 24 h and reached a maximum at 6 h after treatments (500-fold). Similar to *CsLOX2*, *CsLOX1* was also induced by ABA, ACC, cold, NaCl and KCl. However, the induced effects of cold and KCl on *CsLOX1* were not comparable to that of *CsLOX2* (Figure 4).

As shown in Figure 5, for *CsLOX4*, MeJA had the greatest effect on its mRNA accumulation, whereas ABA, ACC, wounding and KCl induced its up-regulation slightly. While expression of *CsLOX8* was largely up-regulated with MeJA and induced by wounding, and also synergistically induced by ACC greatly, suggesting *CsLOX8* may be also involved in signal crosstalk between MeJA and ethylene. Meanwhile, transcripts of *CsLOX8* increased in response to ABA, NaCl and KCl at the later stage of these treatments.

Interestingly, *CsLOX9*, a type-1 LOX with 9-LOX activey biochemically, together with *AtLOX5*, which is involved in lateral root growth [36,45], had relatively stable expressions to exogenous treatments and were only significantly down-regulated by cold and ACC (Figure 6). Similar to the report on a citrus 9-lipoxygenase that could be specifically induced by salt stress, other than osmotic stress [46], *CsLOX10* only responded to NaCl treatment and was significantly up-regulated by approximately 50-fold at 12 hr after treatment (Figure 6).

For the six predicted type-2 LOX genes (*CsLOX16, 17, 19, 20, 22* and *23*), as shown in Figures 7–9, all these genes were largely up-regulated by MeJA, and induced simultaneously by wounding. Four genes (*CsLOX 16*, *17*, *19* and *23*) were also found to be up-regulated at least 6-fold at 6 hr after ACC treatment, whereas *CsLOX20* and *CsLOX22* were down-regulated by ACC up to 100-fold at 6 hr after treatment. Furthermore, it was shown that five genes (*CsLOX17*, *19*, *20*, *22* and *23*) were positively regulated by ABA, cold, NaCl and KCl treatments, except that *CsLOX16* was almost no response to these treatments.

## 3. Discussion

### 3.1. Cucumber LOX in Fruit Development

Previous studies have shown that in various plant species, *LOX* were found to be involved in fruit development and ripening for membrane deterioration, peroxidation of polyunsaturated fatty acids and oxylipin accumulation, resulting in the cell breakdown, the loss of compartmentation and the production of flavor volatiles [8–21]. The sequence analysis of the cucumber (*Cucumis sativus*) genome identified the presence of at least 23 LOX-like sequences, in which nine belong to type-1 LOX, 13 belong to type-2 LOX and one orphan gene [38]. Huang *et al*. suggested that the high level synthesis of NDE in the cucumber might be one of the reasons why cucumber *LOX* gene family expands so much [37].

In our current study, it has been shown that the expressions of 13 *LOX* genes are widely distributed within cucumber organs and tissues (Figure 1 and supplemental Table 1). Among the 13 *LOX* genes, transcripts of 12 genes (*CsLOX1*, *2*, *4*, *8*, *9*, *10*, *16*, *17*, *19*, *20*, *22* and *23*) accumulated in cucumber fruit and were expressed differentially during fruit development (Figures 2,3). Our results indicated that these 12 *LOX* genes could be divided into four expression patterns during fruit development: I “low-high-low” (including *CsLOX1*, *CsLOX2*, *CsLOX4*, *CsLOX8*, *CsLOX10*, *CsLOX19* and *CsLOX20*), II “low-high” (including *CsLOX9* and *CsLOX23*), III “high-low” (including *CsLOX16* and *CsLOX22*) and IV “high-low-high-low” (including *CsLOX17*) (Figure 2). According to their expression during fruit development, we suggested that *LOX* genes in pattern I might be involved in fruit cell development after anthesis, and *CsLOX2*, the only one which showed peak values from 5 to 7 DAA, might participate in aroma production, as the flavor volatile NDE was reported to increase suddenly in fruit on 5 DAA [47]. The *LOX* genes in pattern II would have roles in cell degradation and senescence because they kept increasing during fruit development and ripening. The *LOX* genes in pattern III were thought to be involved in ovary development before anthesis, whereas genes in pattern IV were predicted to participate in fruit growth both before and after anthesis. However, there is a great deal of work to do before the specific functions of these cucumber *LOX* genes are confirmed.

The comparative studies of the distributions of *LOX* transcripts within cucumber fruit fractions on 5 DAA also suggested their various functions (Figure 3). However, to further our understanding of cucumber *LOX* functions within developing fruit, this analysis must be repeated across all stages of cucumber fruit development to determine whether the distributions of *LOX* gene transcripts change in developing fruit.

### 3.2. Cucumber *LOX* Gene Expressions in Response to Plant-Growth Regulators and Abiotic Stresses

The regulation of *LOX* gene expressions by different effectors, such as JA, ABA, and ethylene, and by different forms of stress, such as wounding, cold and salt stress, have been revealed in some plant species in recent decades [3,23,27,46,48,49]. In our current study, cucumber *LOX* transcripts exhibited differential accumulation or downregulation in response to plant-growth regulators (MeJA, ACC, and ABA) and abiotic stresses (wounding, chilling, NaCl and KCl) (Figures 4–9).

The induction of *LOX* transcripts has been observed in several species after mechanical wounding. The function of LOX proteins in wounding seems to be related to the synthesis of a number of different compounds that can alleviate the effects from harm. JA is one of most important signaling molecules in wound response [48,50,51], and LOX proteins are involved in JA synthesis [24]. In most cases, wound-induced *LOX* genes are also induced by exogenous JA [3].

Our results showed that 10 out of 12 fruit *LOX* genes (*CsLOX1*, *2*, *4*, *8*, *16*, *17*, *19*, *20*, *22* and *23*) were inducible by wounding and were simultaneously largely up-regulated with MeJA, of which eight (*CsLOX1*, *2*, *4*, *8*, *16*, *17*, *19* and *23*) were also positively induced by ethylene, and the transcripts of two (*CsLOX20* and *22*) were downregulated by ethylene treatment (Figures 4–9). Ethylene is a hormone playing vital roles in fruit softening and ripening, and, in turn, superoxide free radicals and hydroperoxides produced by LOX protein during fruit softening and ripening may participate in ethylene production [52,53]. We infer that besides their role in synthesis of compounds alleviating damage from wounding, these ethylene-responsive cucumber *LOX* genes might also be involved in feedback loops of ethylene stimulation and production during fruit development.

ABA was demonstrated to be a key regulator of plant in responses to abiotic stresses such as chilling and salt stress [54]. ABA treatment, cold and salt stresses are known to induce *LOX* mRNA accumulation. Our study shows that some of the cucumber *LOX* genes, including *CsLOX1*, *2*, *8*, *17*, *19*, *20*, *22* and *23*, were up-regulated by ABA, cold and salt treatments; however, we observed that the time points at which these genes reached the maximum in response to cold, NaCl and KCl (3–6 h) were earlier than in response to ABA (12–24 h) (Figures 4,5,7–9). This may be a result of the more direct effect of cold, NaCl and KCl stress on membrane deterioration and the immediate induction of LOX activity [55]. However, because salt-stress-induced ABA accumulation is more sensitively triggered in roots than in shoots [56], to demonstrate the inference above, ABA should be supplied by roots just like the NaCl and KCl treatments. It would be interesting to address the above questions in the near future.

Meanwhile, we found that KCl exhibited more significant effect on the mRNA accumulation of *CsLOX2* compared to that of NaCl (Figure 4). It is well known that except the effect of osmotic stress, in many vegetables potassium (K^+^) fertigation improves fruit quality, such as flavor synthesis. Moreover, in the research *CsLOX2*, a type-1 LOX predicted with 9-LOX activity, was demonstrated to mainly express in endocarp and pulp, and show sudden up-regulation in cucumber fruit from 5 to 7 DAA. Therefore, the results suggested a role of *CsLOX2* in the formation of cucumber flavor NDE.

## 4. Methods

### 4.1. Plant Materials and Stress Treatments

After seed germination, cucumber (*Cucumis sativus*) cv Line 9930 (a cultivar of northern China) plants were raised and grown in pots containing mixed peatmoss and vermiculite (v/v = 1:1) in a greenhouse in the Institute of Vegetables and Flowers. They were then moved into a growth chamber kept at 28 °C/22 °C (day/night) with a 12 h photoperiod at a photosynthetic photon flux of 250 μmol m^−2^s^−1^, and the relative humidity was 60%–70%. When plants reached two expanded true leaves, ABA (100 mM), MeJA (100 mM), ACC (100 μM) (Sigma, St. Louis, MO) and water were applied to the foliage of selected uniform seedlings. Plants treated with MeJA and ACC were tightly sealed in a plastic bag. For salinity treatments, seedlings were removed from soil and subjected to 200 mM NaCl or 200 mM KCl solutions. For wounding experiments, leaves were pricked with a needle. For cold treatment, cucumber plants were exposed to another growth chamber kept at 4 °C under a 12 h photoperiod with the same conditions described above. These cucumber plants were sampled at various time points after treatment, frozen in liquid nitrogen, and stored at −80°C until used for RNA isolation.

Cucumber (*Cucumis sativus*) cv Line 9930 plants grown in the greenhouse of the Institute of Vegetables and Flowers were used for tissue-specific expression analysis. The roots, stems, tendril, cotyledons, true leaves, male flowers, female flowers, ovaries and fruits of mature plants were collected separately for RNA isolation. The fruits on day 0, 1, 3, 5, 7, 10, 12 and 14 after anthesis (DAA) were also sampled. The fruits on 5 DAA were also divided into exocarp, endocarp and pulp respectively. These samples were frozen quickly in liquid nitrogen, and stored at −80 °C for further analysis.

### 4.2. RNA Isolation and Semi-Quantitative RT-PCR

Total RNA was isolated from various tissues of plants using the Trizol reagent (Invitrogen) according to the manufacturer’s instructions. RNA concentration was determined with a NanoDrop ND-1000 photospectrometer. Reverse transcription was performed with 5 μg of total RNA using the M-MLV reverse transcriptase (Promega) according to the manufacturer’s instructions. The cucumber annotated (predicted) genes and proteins were obtained from the Cucumber Genome Sequencing Project, which we participated in. These cucumber *LOX* gene sequences were downloaded from the Cucumber Genome DataBase [38], and specific primers were designed for RT-PCR and real-time PCR (supplement Table 3). A cucumber ubiquitin gene *CsUBQ* (Csa000874) was used as a control. The productions of RT reaction were used as templates for PCR analysis. The number of cycles of PCR for *LOX* genes was 30, while that for *CsUBQ* was 25.

### 4.3. Quantitative RT-PCR Analysis

Quantitative real-time PCR was performed following the protocol of the Perfect Real-time PCR kit (TaKaRa) on the Applied BioSystems 7500 Real Time PCR System (Applied BioSystems). Aliquots of the products of RT reaction were used as templates for real-time PCR. For relative quantification, *CsUBQ* (Csa000874) (for the quantitation of gene expression during development) and *CsACT* (Csa017310) (for the quantitation of gene expression during abiotic stresses) genes were detected as an internal reference, and the 2^−ΔΔCt^ method was used.

## 5. Conclusions

We identified the expressions of 13 out of 23 *CsLOX* genes during vegetative and reproductive stages of development. The results indicated that these 13 *CsLOX* genes were expressed with tissue-specific or preferential accumulation, of which 12 were differentially expressed during fruit development and exhibited different patterns in the different fractions of cucumber fruit. The expression analysis of these 12 cucumber *LOX* genes in response to abiotic stress and plant growth regulator treatments revealed their differential expression in response to more than one treatment, indicating their diverse functions in abiotic stress and hormone response and suggesting a potential mechanism to regulate LOX activity for improving cucumber fruit quality. According to our results, we suggest that *CsLOX2*, a type-1 LOX predicted to be 9-LOX, might be involved in the formation of cucumber flavor NDE.
